# Antioxidant and Antidiabetic Properties of a Thinned-Nectarine-Based Nanoformulation in a Pancreatic β-Cell Line

**DOI:** 10.3390/antiox13010063

**Published:** 2023-12-31

**Authors:** Elisabetta Schiano, Ettore Novellino, Marta María Gámez Fernández, Helena Tiekou Lorinczova, Gian Carlo Tenore, Fortuna Iannuzzo, Vinood B. Patel, Satyanarayana Somavarapu, Mohammed Gulrez Zariwala

**Affiliations:** 1 Department of Pharmacy, University of Naples Federico II, via Domenico Montesano 59, 80131 Naples, Italy; elisabetta.schiano@unina.it (E.S.); giancarlo.tenore@unina.it (G.C.T.); fortuna.iannuzzo@unina.it (F.I.); 2 Healthcare Food Research Center, Inventia Biotech s.r.l., Strada Statale Sannitica KM 20.700, 81020 Caserta, Italy; 3 Department of Medicine and Surgery, Catholic University of the Sacred Heart, 00168 Rome, Italy; ettore.novellino@unicatt.it; 4 Centre for Nutraceuticals, School of Life Sciences, University of Westminster, 115 New Cavendish Street, London W1W 6UW, UK; m.gamezfernandez@westminster.ac.uk (M.M.G.F.); h.tiekoulorinczova@westminster.ac.uk (H.T.L.); v.b.patel@westminster.ac.uk (V.B.P.); 5 Department of Pharmaceutics, UCL School of Pharmacy, 29-39 Brunswick Square, London WC1N 1AX, UK; s.somavarapu@ucl.ac.uk

**Keywords:** agro-food waste products, polyphenols, bioactive compounds, abscisic acid, nanoformulations, pancreatic β-cells

## Abstract

Pancreatic β-cells play a crucial role in maintaining glucose homeostasis, although they are susceptible to oxidative damage, which can ultimately impair their functionality. Thinned nectarines (TNs) have gained increasing interest due to their high polyphenol and abscisic acid (ABA) content, both of which possess antidiabetic properties. Nevertheless, the efficacy of these bioactive compounds may be compromised by limited stability and bioavailability in vivo. This study aimed to develop nanoformulations (NFs) containing pure ABA or a TN extract (TNE) at an equivalent ABA concentration. Subsequently, the insulinotropic and antioxidant potential of the NFs and their unformulated (free) forms were compared in MIN-6 pancreatic cells exposed to varying glucose (5.5 mM and 20 mM) and iron (100 µM) concentrations. NF-TNE treatment exhibited enhanced antioxidant activity compared to free TNE, while ABA-based groups showed no significant antioxidant activity. Moreover, MIN6 cells incubated with both high glucose and iron levels demonstrated significantly higher insulin AUC levels after treatment with all samples, with NF-TNE displaying the most pronounced effect. In conclusion, these results highlight the additional beneficial potential of TNE due to the synergistic combination of bioactive compounds and demonstrate the significant advantage of using a nanoformulation approach to further increase the benefits of this and similar phytobioactive molecules.

## 1. Introduction

Pancreatic β-cells play a pivotal role in the management of glucose homeostasis by synthesizing and secreting insulin at appropriate rates. At low levels, oxidative stress is essential for normal β-cell function and has a role in different activities, such as the stimulation of basal insulin secretion [1]. However, due to the relatively low expression of antioxidant enzymes (e.g., catalase and glutathione peroxidase) [2], these cells are profoundly susceptible to oxidative damage upon sustained insult [3]. In particular, reactive oxygen species (ROS) are key mediators for nucleic acids, lipids, and protein damage, resulting in undesirable biochemical and functional modifications [4]. Sustained excessive iron and glucose exposure are key mediators of oxidative stress in pancreatic β-cells, ultimately leading to mitochondrial and cellular membrane damage and disruption [5]. High amounts of free ferrous iron lead to ROS production via the Fenton and Haber–Weiss reactions, involving the production of hydroxyl radical (^•^OH). These free radicals can react rapidly with biological molecules, causing significant damage to cellular components and dysfunctions including insulin resistance and eventual β-cell failure [1]. In this context, recent evidence demonstrated that high iron exposure (100 µM) can result in a marked decrease in MIN6 insulin secretory capacity as well as cellular insulin content. From a mechanistic point of view, the perturbations in mitochondrial functionality related to iron-induced oxidative stress is associated with a reduction in MIN6 β-cell insulin secretion, indicating a fundamental role for iron overload in the onset and progression of T2DM [6]. 

The potential efficacy of bioactive compounds of natural origin in the treatment of diabetes through the ability to control blood glucose levels to different degrees and through varying mechanisms of action has been well described in the scientific literature [7]. Interestingly, immature fruits derived from fruit thinning, the manual elimination of fruitlets, have gained increasing attention for nutraceutical applications as food byproducts rich in bioactive compounds with potential health benefits [8]. Specifically, recent findings suggested that thinned nectarines (TNs) (*Prunus persica* L.) are promising for the management of diabetes due to their high content of polyphenols and abscisic acid (ABA) [9,10]. ABA is a naturally occurring plant hormone found at the immature stage in various fruits, including nectarines [11]. Moreover, several studies have also demonstrated a role for ABA as an endogenous hormone exhibiting antidiabetic properties in humans by enhancing glucose uptake and improving insulin sensitivity [12,13]. Among plant-derived compounds, polyphenols have also demonstrated potential in diabetes control [14]. These molecules exert antioxidant and anti-inflammatory properties that can help regulate glycemic levels and improve insulin resistance [15]. Notably, TNs have been shown to contain high levels of polyphenols compared to their mature counterparts, making them an intriguing option for individuals with diabetes [9,16,17]. Overall, the combined presence of abscisic acid and polyphenols in TNs suggests a synergistic effect in terms of their potential antidiabetic properties. 

Although several natural compounds have shown promising effects, their application has often been limited by issues related to their relatively low stability, bioavailability, and efficacy at the site of action [18]. In this context, nanotechnology-based formulations have proven to be beneficial in overcoming these limitations and enhancing the potential of bioactive molecules [18,19]. Polymeric micelles (PMs) are such nanoparticle delivery systems and have been used as drug carriers as they provide a suitable platform for the incorporation of active ingredients. In this regard, several studies successfully demonstrated the role of these vesicles as carriers for hydrophobic molecules [20]. Among different polymeric micelles, the polyethylene glycol-grafted 1,2-distearoyl-sn-glycero-3-phosphatidylethanolamine (DSPE-PEG) stands out due to its ability to form micelles in aqueous environments rather than creating bilayered structures [21]. Ascorbyl palmitate (AP), a lipophilic derivative of ascorbic acid, is commonly utilized in the cosmetic industry. Due to its hydrophobic nature, AP spontaneously combines with the amphiphilic derivative DSPE-PEG to form vesicular structures in an aqueous environment [21,22]. 

Based on these considerations, this study aimed to develop, for the first time, nanoformulations (NF) containing a pure standard of ABA or a thinned nectarine extract (TNE) at the same ABA concentration using DSPE-PEG-AP as the nanocarrier system. Moreover, the insulinotropic and antioxidant potential of these nanoformulated compounds and their unformulated forms were tested and compared in MIN-6 pancreatic cells incubated with a combination of different glucose (5.5 mM and 20 mM) and iron (100 µM) concentrations. 

## 2. Materials and Methods

### 2.1. Reagents and Materials

All chemicals and reagents used for the HPLC-DAD analysis were either analytical-reagent or HPLC grade. The water was treated in a Milli-Q water purification system (Millipore, Bedford, MA, USA) before use. (±)-2-*cis*-4-trans-abscisic acid (ABA), 2′,7′-Dichlorofluorescein diacetate (DCFH-DA), ethanol, methanol, acetonitrile, and formic acid were all purchased from Sigma-Aldrich (Milano, Italy). Iron(II) sulfate heptahydrate (FeSO_4_) was used for all experiments involving the use of iron as a prooxidant agent. All chemicals for cell culture experiments were of cell-culture grade and purchased from Sigma-Aldrich (Dorset, UK) unless otherwise stated. Protease Inhibitor Cocktail (PIC, catalog no. P8340), dimethyl sulfoxide (DMSO), Thiazolyl, and Blue Tetrazolium Blue (MTT) were obtained from Sigma-Aldrich (Dorset, UK). Fisher Scientific (Loughborough, UK) supplied Minimum Essential Medium (MEM), Dulbecco’s modified Eagle’s medium (DMEM) Glutamax^®^, fetal bovine serum (FBS), and 100× antibiotic–antimycotic solution. The 6- and 12-well cell culture dishes, 96-well microtiter plates, and flasks were purchased from Nunc (Roskilde, Denmark). A mouse insulin ELISA kit was supplied by Mercodia (Cat No. 10-1247-01—Mercodia, D.B.A., Milan, Italy). DSPE-PEG was purchased from Lipoid (Steinhausen, Switzerland), and ascorbyl palmitate was from Sigma-Aldrich (Dorset, UK). Experimental reagents were prepared using ultrapure water (resistivity of 18.2 MΩ cm).

### 2.2. Preparation of Thinned Nectarine Extract 

TNs were harvested in June 2022 at the orchards of “Giaccio Frutta” society (Vitulazio, Caserta, Italy, 41°10′ N–14°13′ E) about one month after full bloom in concomitance with the fruit thinning stage. The whole fruits (pulp, peel, and seed) were frozen at −20 °C, lyophilized, and ground to obtain a homogenous powder. With regards to the extraction procedure, a previous method reported by Schiano et al. was used with slight modifications [23]. Briefly, 2.5 g of homogenized sample was suspended in 25 mL of a 100% ethanolic solution and thoroughly vortexed for 1 min. The suspension was then sonicated for 10 min using a VWR Ultrasonic cleaner bath USC300T (VWR International Limited, Lutterworth, UK) and stirred lightly for 40 min at 25°, 600 RPM speed. After centrifugation (10 min, 9000× *g*, 25°), the supernatant was decanted, filtered with a 0.22 μm nylon filter (CellTreat, Shirley, MA, USA), and evaporated using a rotary evaporator (Hei-VAP Advantage Rotary Evaporator, Heidolph, Germany). The ABA content of the dried extract was evaluated as reported in the following section. For all experiments described below, the dried extract was dissolved with a defined volume of media (MEM) in order to obtain the equivalent ABA concentrations contained in free ABA and NF-ABA samples.

### 2.3. HPLC-DAD Analysis of Abscisic Acid

The chromatographic system utilized was a Jasco Extrema LC-4000 (Jasco Inc., Easton, MD, USA) equipped with the following modules: a vacuum degasser, an autoinjector, a quaternary pump, a column oven, and a diode array detector photodiode array detector (DAD). This setup was used for the chromatographic determination of ABA content in TNE based on a previous method, with slight modifications [24]. The chosen column was a Kinetex^®^ C_18_ column 100A (250 mm × 4.6 mm i.d., 5 μm) from Phenomenex (Torrance, CA, USA). The mobile phases consisted of 1% formic acid in water (A) and acetonitrile (B). The elution procedure started with a 3-min hold at 5% of solvent B, followed by a gradient to 75% (B) over 20 min and a 1 min maintenance; the column was then re-equilibrated to the initial conditions for another minute. Separation conditions included a 30 °C column temperature, 20 µL injection volume, and a 1 mL/min flow rate. Abscisic acid detection was monitored at 265 nm, with quantification based on a calibration curve covering 0.1–100 ppm concentrations and triplicate injections at each level.

### 2.4. Evaluation of the Physiochemical Properties of the Nanoformulations

#### 2.4.1. Preparation of Nanoformulated Samples

The preparation of DSPE-PEG or DSPE-PEG ascorbyl palmitate nanocarriers was carried out by following the thin-film hydration method, as previously described [21]. Briefly, DSPE-PEG alone or ascorbyl palmitate (AP) and DSPE-PEG were dissolved in methanol and then dried in a round-bottom flask using a rotary evaporator (Hei-VAPAdvantage Rotary Evaporator, Heidolph, Schwabach, Germany) at 60 °C to form a lipid film. This film was then purged with nitrogen gas to remove residual solvent and then hydrated with either an ABA or TNE solution (4 mg/10 mL or 52 mg/5 mL) or distilled water in the case of corresponding blank nanocarrier formulations. In order to remove any unloaded compound, the obtained solution was filtered with a sterile 0.22 μm filter. The nanocarriers were then stored in 10 mL vials at 4 °C until use. Preparations were coded: 1. DSPE-PEG-TNE, 2. DSPE-PEG-ABA, 3. DSPE-PEG-blank, 4. DSPE-PEG-AP-TNE, 5. DSPE-PEG-AP-ABA, and 6. DSPE-PEG-AP-blank. To allow the storage of samples for further analysis, samples were freeze-dried using a Virtis AdVantage 2.0 BenchTop freeze dryer (SP Industries, Bradford, UK). 

#### 2.4.2. Size and Surface Charge of the Nanoformulations

The particle size and surface charge of the NFs were determined using the Zetasizer Nano ZS (Malvern Instruments, Malvern, UK). For these measurements, the nanodispersions were added individually to the ZetaMaster electrophoresis cell to assess their size distribution via photon correlation spectroscopy as mean volume diameters (MVD) and polydispersity index (PDI), carrying out three measurements on each sample. The zeta potential of NF samples was determined similarly using the electrophoretic light-scattering technique (Zetasizer Nano ZS, Malvern Instruments, UK), measuring electrophoretic mobility in triplicate for all samples (Table 1).

#### 2.4.3. Nanoformulation Morphology

Transmission electron microscopy (TEM) was used to analyze the complex NF morphology, including the size and surface characteristics, using an FEI CM 120 BioTwin transmission electron microscope (Philips Electron Optics BV, Eindhoven, The Netherlands) at an acceleration voltage of 120.0 kV. Samples of approximately 40 μL of the nanoparticle dispersion were placed on a Formvar/carbon-coated copper grid, negatively stained with 1% uranyl acetate, and observed at magnifications of 13,500-, 17,500-, 46,000-, and 65,000-times magnification to capture digital images (Figure S1).

#### 2.4.4. Determination of Nanoformulation Loading and Encapsulation Efficiency

HPLC-DAD analysis was utilized to study the drug loading and encapsulation efficiency of NF, following the method previously described in Section 2.3. The percentage of drug loading (DG) and encapsulation efficiency (EE) were calculated based on the following equations:

(1) $$\mathrm{DG}\;\left(\%\right)=\frac{\mathrm{Determined}\;\mathrm{mass}\;\mathrm{of}\;\mathrm{drug}\;\mathrm{within}\;\mathrm{nanocarriers}}{\mathrm{Mass}\;\mathrm{of}\;\mathrm{drug}-\mathrm{loaded}\;\mathrm{nanocarriers}\;}\times 100$$

(2) $$\mathrm{EE}\;\;\left(\%\right)=\frac{\mathrm{Determined}\;\mathrm{mass}\;\mathrm{of}\;\mathrm{drug}\;\mathrm{within}\;\mathrm{nanocarriers}}{\mathrm{Theoretical}\;\mathrm{mass}\;\mathrm{of}\;\mathrm{drug}\;\mathrm{within}\;\mathrm{nanocarriers}\;}\times 100$$

### 2.5. MIN6 Cell Culture 

MIN6 β-cells from mice (originally from Prof. Jun-ichi Miyazaki, Osaka University, Osaka, Japan) were kindly provided by the Beta Cell Group (Department of Diabetes, King’s College London, London, UK) at passage 35. Cells stored at cryogenic temperatures (−196 °C) in liquid nitrogen were reconstituted rapidly by placing them in an incubator with an atmosphere of 95% air and 5% CO_2_ at 37 °C for 2 min. After transferring the cells to a 15 mL centrifuge tube, 5 mL of fresh growth medium was added. The cell suspension was then centrifuged at 5000 RPM for 5 min. The resulting pellet was resuspended in a 75 cm^2^ T-flask containing 10 mL of fresh growth media. MIN6 cells were cultured in a Dulbecco’s Modified Eagle Medium (DMEM)—GlutaMax^®^, pH 7.4, supplemented with 1% antibiotic/antimycotic solution and 10% Fetal Bovine Serum (FBS), and maintained in a 95% air, 5% CO_2_ atmosphere at 37 °C with constant humidity. Cells were seeded in 12-well or 6-well plates for all experimental procedures and reached confluence within five days. At this time, small clusters of cells were formed, indicating that the cells were assembled as physiologically relevant mature pseudoislets and were viable and functional (Figure S2).

### 2.6. Determination of Cell Viability by MTT Assay

To assess any potential cytotoxicity of the testing samples (free ABA, free TNE, NF-ABA, and NF-TNE) on MIN6 beta cells, cells were incubated with each sample at three different ABA concentrations (0.001, 0.01, and 0.1 µM) or media (MEM) for 24, 48, and 72 h. After each incubation time, a volume of 20 µL of 3-(4,5-Dimethylthiazol-2-yl)-2,5-diphenyltetrazolium bromide (MTT) solubilized in sterile Dulbecco’s Phosphate-Buffered Saline (DPBS) was added to the wells at the concentration of 5 mg/mL [19]. After incubation at 37 °C for 4 h, the formazan crystals formed were dissolved in 100 µL of DMSO, with the previous medium aspirated. The plates were ultimately placed on The MaxQ 4000 benchtop orbital shaker (Thermo Fisher Scientific, Loughborough, UK) at 75 rpm for 15 min to ensure the DMSO was mixed well. The absorbance was subsequently measured at 570 nm and compared to the control cells (MEM). 

### 2.7. Cellular Antioxidant Activity (CAA) Assay 

The cellular antioxidant activity (CAA) assay was performed to assess and compare the antioxidant power and cellular accessibility of the formulated and unformulated samples at varying concentrations. To this end, a pre-incubation with a cell-permeable 2′,7′-Dichlorofluorescein diacetate (DCFH-DA) fluorescence probe dye was performed. DCFH-DA is deacetylated by cellular esterase and turns into its fluorescent form DCFH upon oxidation, triggered by the addition of iron (100 µM) as a prooxidant agent. The cellular antioxidant activity was assessed based on the original assay developed by Wolfe and colleagues [25]. MIN6 cells were first cultured in a 96-well black-walled cell culture plate until confluence. Cells were then washed with DPBS and treated with 200 µL of one of the testing samples at the same ABA concentration (0.05 µM) for 1 h at 37 °C. Subsequently, cells were washed with MEM and mixed with 200 µL of DCFH-DA (100 µM) and further incubated for 30 min at 37 °C. Following aspiration, each well was treated with 100 µL of iron at 100 µM. Cell fluorescence was read every 5 min for 1 h at 528 and 485 nm emission and excitation wavelengths, respectively, using a Fluostar Optima Fluorescence Plate Reader (BMG Labtech, Offenburg, Germany). The bioflavonoid Quercetin, ranging from 0 to 2000 μM, was used as a positive control. Based on the hypothesis of the assay, Quercetin and test antioxidant samples inhibit the development of DCF by inhibiting free radical production in a concentration-dependent manner. The CAA units were calculated using the following equations:

(3) $$\mathrm{CAA}\;\mathrm{unit}\;=100-\frac{\mathrm{AUC}\;\mathrm{of}\;\mathrm{the}\;\mathrm{treatment}}{\mathrm{AUC}\;\mathrm{of}\;\mathrm{the}\;\mathrm{control}\;}\times 100\;$$

(4) $$\mathrm{AUC}\;=\left(1+\left(\frac{\mathrm{RFU}1}{\mathrm{RFU}0}\right)+\left(\frac{\mathrm{RFU}2}{\mathrm{RFU}0}\right)\right),$$

where RFU0 is the relative fluorescence value of time zero and RFUx is the relative fluorescence at each time point (e.g., RFU10 is the relative fluorescence value at minute 10). The results of the positive control Quercetin were also reported as IC_50_, while the samples’ antioxidant values were expressed as both CAA units and Quercetin equivalents (QE).

### 2.8. MIN6 Cells Exposure to Glucose and Iron 

Cells were seeded at 75 × 10^4^ cells/cm^2^ for the glucose-stimulated insulin secretion (GSIS) and the iron challenge assay. The day before the experiment, MIN6 cells were iron-depleted with a serum-free MEM media (glucose 5.5 mM, supplemented with 2 mM L-glutamine and 1% antibiotic/antimycotic) and incubated overnight at 37 °C with 5% CO_2_. For the GSIS experiment, MIN6 cells were washed with DPBS and preincubated in a Krebs–Ringer Bicarbonate (KRB) buffer (10 mM HEPES, 119 mM NaCl, 1.19 mM KH_2_PO_4_, 1.19 mM MgSO_4_·7H_2_O, 4.74 mM KCl, 25 mM NaHCO_3_, 2.54 mM CaCl·6H_2_O, pH 7.4, and 1% BSA) containing 1.1 mM glucose (basal level) for 2.5 h. For the iron challenge assay, cells were incubated in a KRB buffer containing a high iron concentration (100 µM) for 2.5 h. Afterward, an aliquot of the supernatant was collected for T0 analysis and cells were then preincubated for 30 min with the different sample treatments (free ABA, free TNE, NF-ABA, NF-TNE). A negative control (represented by MEM) was also tested. Finally, media were aspirated from all the wells, followed by 2 h exposure to KRB solution at two glucose concentrations, 5.5 mM (physiological glucose concentration) and 20 mM (supraphysiological glucose concentration). Immediately after the exposure to glucose, the supernatants were collected at different time points for analysis (i.e., T5, T10, T30, T60 min), while cells were lysed at the end of the incubation time (60 min). Table S1 reports the list of samples at different glucose and iron concentrations. All supernatants and cell lysates were aliquoted and stored at −20° C. 

### 2.9. Quantification of Intracellular Total Iron 

Intracellular total iron content was quantified using an optimized FerroZine™-based iron protocol developed by previous authors, with slight modifications [26]. Briefly, 200 µL of 0.1 M HCl solution was added to tubes containing 200 µL of the sample, while 200 µL of 50 mM NaOH solution was used for tubes containing 200 µL of each standard of iron (from 0 to 120 µM). A blank solution contained 400 µL of 50 mM NaOH. Subsequently, a volume of 200 µL of the freshly prepared iron-releasing agent, consisting of an equal volume of 1.4 M HCl and 4.5% (*w*/*v*) KMnO_4_ solution, was added to each tube to promote the iron release from proteins, including ferritin. Samples were incubated for 2 h in a 60 °C dry bath within a fume hood and then cooled to room temperature for 10 min. Afterward, a volume of 60 µL of the iron detection reagent (6.5 mM FerroZine™, 2.5 M ammonium acetate, and 1 M ascorbic acid dissolved in water) was mixed into each tube and samples were incubated at 25° for 60 min. Lastly, a volume of 200 µL from standard and sample tubes was placed in duplicate into a 96-well plate and the color development was spectrometrically monitored by measuring the absorbance at 550 nm using a microplate reader (Fluostar Optima Plate Reader (BMG Labtech, Offenburg, Germany)). Intracellular iron concentration was determined by normalizing the obtained results against the total protein content of each well evaluated by the BCA (bicinchoninic acid) assay. 

### 2.10. Evaluation of Insulin Secretion

The measurement of insulin secretion is considered pivotal for the investigation of β-cell function. To this aim, supernatants were obtained from MIN6 cells exposed to different concentrations of glucose and iron at various time points (T0, T5, T10, T30, T60 min) to determine the temporal profile of insulin secretion under these conditions and were then analyzed by insulin ELISA assay. Briefly, a volume of 10 µL of standards and samples was transferred into a 96-well plate in duplicate followed by the incubation steps, as described by the manufacturer’s protocol (Cat No. 10-1247-01—Mercodia, D.B.A., Milan, Italy). Subsequently, the absorbance was measured at 450 nm using a microplate reader.

### 2.11. Statistical Analysis

For all the experiments, the average was calculated from at least four replicates per condition, with data expressed as the mean ± standard error of the mean (SEM) unless otherwise stated. The MTT results were analyzed using a one-way analysis of variance (ANOVA) followed by Dunnett’s post hoc test and a two-way ANOVA followed by Sidak’s post hoc test. Total iron quantification was examined using a two-way ANOVA test followed by Sidak’s post hoc test. CAA results were evaluated with a two-tailed Student’s *t*-test, while ELISA results were analyzed using a two-way ANOVA test followed by Tukey’s multiple comparisons post hoc test (PRISM software package, Version 8, Graphpad Software Inc., San Diego, CA, USA). 

## 3. Results

### 3.1. Size, Charge, and Loading Efficiency of Nanoformulations

In all cases, NF samples were found to be in the nanometer size range (<200 nm), as confirmed by Zetasizer analysis and morphological assessments. These data are in line with the project objectives, as the aim was to create nanocarriers loaded with bioactive compounds, as particles in this size range may exhibit enhanced cellular uptake and longer drug retention time in the bloodstream by potentially avoiding phagocytic clearance. The incorporation of AP resulted in an increase in the particle size of the NF, although in all cases the bioactive-loaded NFs were smaller compared to the corresponding blanks. The zeta potential results do not differ significantly between the formulations but show a shift towards a positive charge in the case of NF-ABA and NF-TNE compared to the blank. Overall, these data are also in general agreement with the results of previous studies using a similar carrier system for iron delivery [21]. Furthermore, the results of the loading efficiency of the nanocarriers revealed a very high encapsulation of 95% and 99% in the case of NF-ABA and NF-TNE, respectively. These results demonstrate the successful incorporation of both ABA and TNE into the carrier systems. High encapsulation efficiency allows the delivery of a higher payload with a lower dose and is a desirable property in formulation design. The observed high encapsulation efficiency demonstrates the suitability of the carrier system and the formulation method used for the entrapment of both the bioactive ABA and TNE.

### 3.2. Assessment of Cell Viability

**Figure 1:**
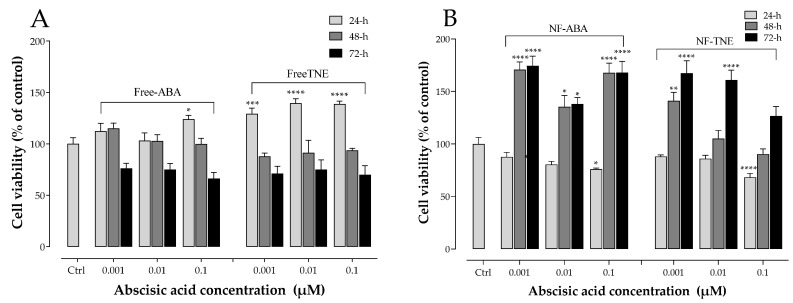
MTT analysis on MIN6 cell viability at 24, 48, and 72 h. (**A**) Cells treated with free ABA and free TNE; (**B**) cells treated with NF-ABA (ascorbyl palmitate/DSPE-PEG nanoformulated abscisic acid) and NF-TNE (ascorbyl palmitate/DSPE-PEG nanoformulated thinned nectarine extract); values are presented as means ± S.D. of 4 replicates. Cells incubated with MEM were regarded as the control in the analysis; data were analyzed with a one-way ANOVA followed by Dunnett’s post hoc test; * p ≤ 0.05, ** p ≤ 0.01, *** p ≤ 0.001, **** p < 0.0001, significantly different from the control group. ABA, abscisic acid; Ctrl, control; TNE, thinned nectarine extract.

The results shown in Figure 1 demonstrate the effects of tested samples (free ABA, free TNE, NF-ABA, and NF-TNE) at three different ABA concentrations (0.001, 0.01, and 0.1 µM) on MIN6 cell viability at 24, 48, and 72 h. As evident, all cells exposed at the ABA concentrations of 0.001 and 0.01 µM did not show reduced cell viability, whereas a 0.1 µM ABA concentration caused a significant reduction in cell survival in the free ABA (*p <* 0.05 vs. control at 72 h), NF-ABA (*p <* 0.001 vs. control at 24 h), and NF-TNE (*p <* 0.0001 vs. control at 24 h) groups. Interestingly, the 24 h incubation with free TNE was demonstrated to significantly increase cell viability for all ABA concentrations tested (*p <* 0.001 vs. control). Moreover, a clear trend of increase in MIN6 cell survival was observed over the 72 h incubation period in both the NF-ABA and NF-TNE groups at most of the concentrations tested, compared to the control.

**Figure 2:**
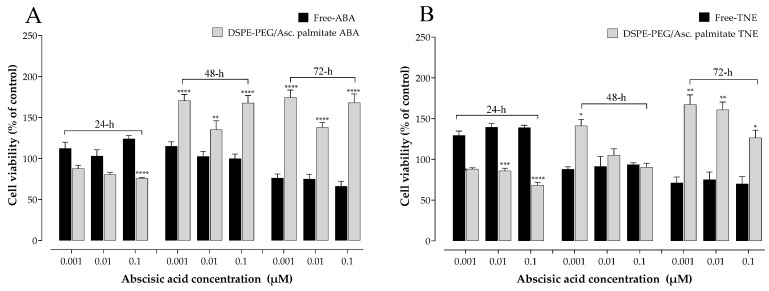
MTT results of 24, 48, and 72 h pre-treatment with (**A**) DSPE-PEG/ascorbyl palmitate nanoformulated ABA and free ABA, (**B**) DSPE-PEG/ascorbyl palmitate nanoformulated TNE and free TNE on MIN6 cells; values are presented as means ± SEM of 4 replicates. Cells incubated with MEM were regarded as the control in the analysis; data were analyzed with two-way ANOVA followed by Sidak’s post hoc test; * p < 0.05, ** p < 0.01, *** p < 0.001, **** p < 0.0001 vs. not nanoformulated sample at the same treatment concentrations; ABA, abscisic acid; Ctrl, control; TNE, thinned nectarine extract; DSPE-PEG, 1,2-distearoyl-sn-glycero-3-phosphatidylethanolamine-N-(polyethylene glycol-2000).

Similarly, the increased cell viability observed in the NF samples was significantly higher compared to free samples at the same ABA concentrations (Figure 2). Besides the novel results in terms of ameliorated cell survival for the nanoformulated samples, these data indicated that concentrations lower than 0.1 µM ABA did not compromise β-cell viability in all cases. Based on the outcomes of these experiments, an intermediate ABA concentration of 0.05 µM was therefore selected for further experiments involving the investigation of the role of the various samples in regulating MIN6 cell function, as this concentration can be regarded as the consensus that may evoke functional effects without compromising cell viability.

### 3.3. Determination of Cellular Antioxidant Activity

**Figure 3:**
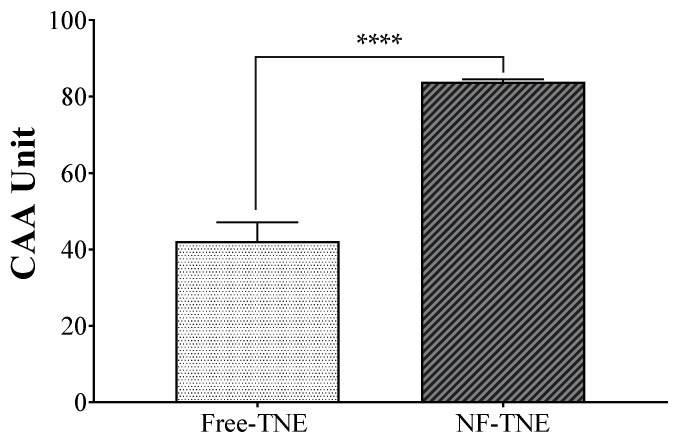
Cellular antioxidant activity (CAA) assay results of nanoformulated and unformulated TNE. Values are presented as the mean ± S.D. of 6 replicates; data were analyzed with a two-tailed Student’s *t*-test; **** p < 0.0001, significantly different from non-nanoformulated samples at the same treatment concentration (ABA 0.05 µM); NF-TNE, ascorbyl palmitate/DSPE-PEG nanoformulated TNE; TNE, thinned nectarine extract.

As reported in the Materials and Methods section, the bioflavonoid Quercetin, ranging from 0 to 2000 μM, was used as a positive control. This latter was shown to exert cellular antioxidant activity in a concentration-dependent manner, with an IC_50_ value of 483 μM (Figure S3). Furthermore, free ABA did not counteract oxidative stress, while the results obtained with TNE treatment showed the ability of this vegetal extract to exert cellular antioxidant properties when cells endure oxidative stress (CAA units: 42.17 ± 4.92; Quercetin equivalents: 483 µM). However, as shown in Figure 3, NF-TNE showed significantly higher antioxidant activity compared with the free TNE sample (CAA units: 83.81 ± 0.74; Quercetin equivalents: 1589 µM; *p <* 0.001 vs. free TNE).

### 3.4. Evaluation of Total Cellular Iron Content

**Figure 4:**
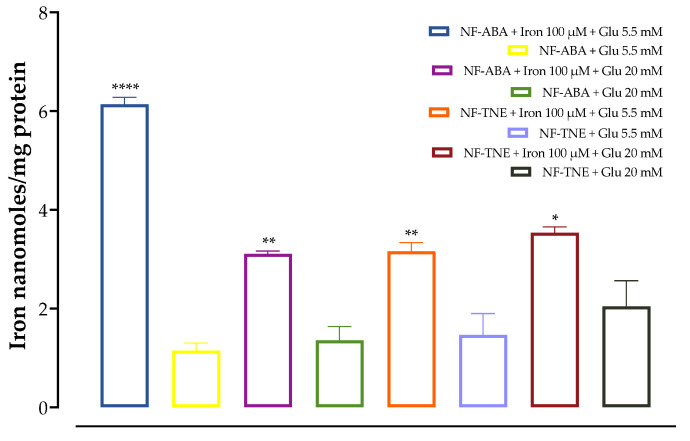
Effects of iron (100 μM) preincubation in MIN6 cells. The data represent mean ± SEM, *n* = 4. Data were analyzed with one-way ANOVA followed by Sidak’s post hoc test; * *p* ≤ 0.05, ** *p* ≤ 0.01, **** *p* ≤ 0.0001 significantly different from samples incubated without iron at the same glucose concentration. Abbreviations: Glu, glucose; NF-ABA, ascorbyl palmitate/DSPE-PEG nanoformulated abscisic acid; NF-TNE, ascorbyl palmitate/DSPE-PEG nanoformulated thinned nectarine extract.

**Figure 5:**
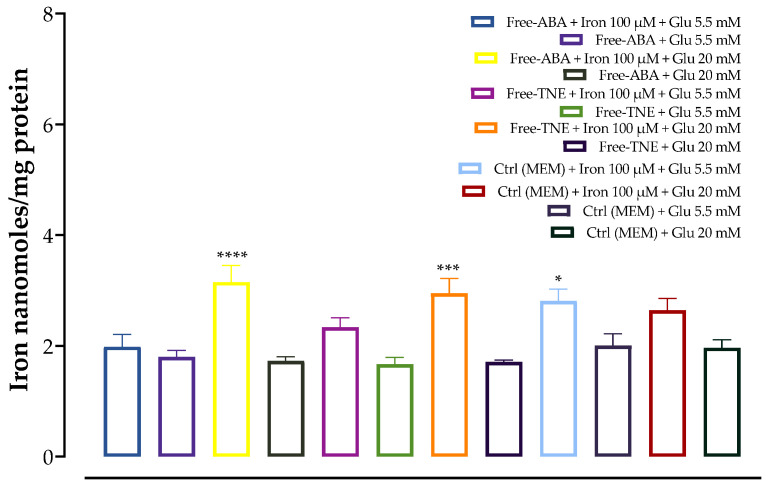
Effects of iron (100 μM) preincubation in MIN6 cells. The data represent the mean ± SEM, *n* = 4. Data were analyzed with one-way ANOVA followed by Sidak’s post hoc test; * p ≤ 0.05, *** p ≤ 0.001, **** p ≤ 0.0001 significantly different from samples incubated without iron at the same glucose concentration. Abbreviations: ABA, abscisic acid; Ctrl, control (MEM); TNE, thinned nectarine extract.

For the iron challenge assay, MIN6 cells were pre-incubated in the presence of 100 µM iron before the addition of glucose at both physiological (5.5 mM) and supraphysiological (20 mM) levels. The evaluation of total iron content in cell lysates allows us to assess if exposure to high iron levels results in a proportionally higher accumulation of iron in MIN6 cells. In this regard, as shown in Figure 4 and Figure 5, a greater iron content was exhibited in all cells pre-incubated with this element, compared to cells incubated with the same glucose concentration without iron. This increase was found to be significant for almost all the samples tested. The highest iron content was observed upon treatment with 100 µM iron and at 5.5 mM glucose in cells treated with NF-ABA, demonstrating a 5-fold increase compared to the same condition without iron incubation. Thus, cellular iron levels were shown to be increased in all cases, demonstrating that iron uptake increased in response to iron exposure, and the presence of the nanoformulation system did not adversely interfere with cellular iron uptake.

### 3.5. Effects of Glucose and Iron on MIN6 Cell Insulin Secretion 

**Figure 6:**
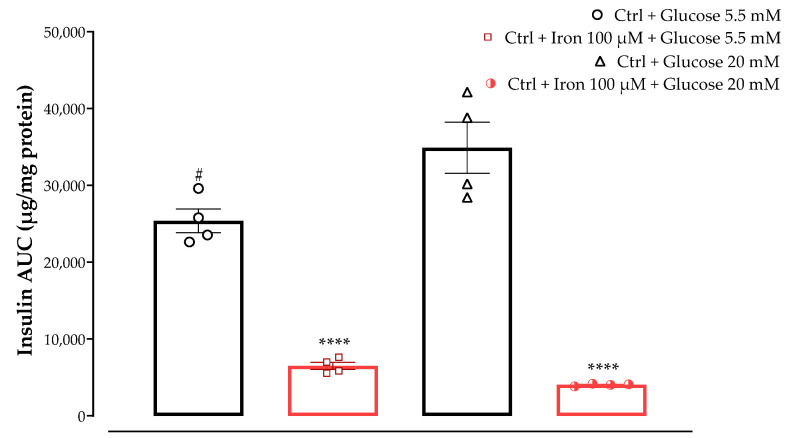
The effect of treatment with two different concentrations of glucose (5 mM and 20 mM) w/wo iron (100 µM) on MIN6 cells insulin secretion. The data represent the mean ± SEM, *n* = 4. Data were analyzed with one-way ANOVA followed by Tukey’s multiple comparison test; **** *p* ≤ 0.0001 significantly different from Ctrl samples incubated with the same glucose concentrations without iron; # *p* ≤ 0.05 significantly different from Ctrl + Glucose 20 mM. Abbreviations: AUC, area under the curve; Ctrl, control (MEM).

The first result that can be highlighted from the last set of experiments is the varying insulin response observed after incubation with two different glucose concentrations (5.5 mM and 20 mM) and the combination of glucose and iron exposure in our cellular model. As shown in Figure 6, a significant increase in insulin AUC was observed in the Ctrl group incubated with 20 mM glucose, compared to the group incubated with 5.5 mM glucose (*p <* 0.05). Of note, pre-incubation with high levels of iron (100 µM) dramatically decreased insulin secretion in the Ctrl groups at both glucose concentrations, suggesting that treatment with this prooxidant agent was responsible for the functional disruption of MIN6 cells subjected to the iron-challenging experiments. As reported in the following sections, the reduction in insulin AUC observed after iron incubation was reproducible for all conditions.

### 3.6. Assessment of Insulin Secretion following NF Exposure

**Figure 7:**
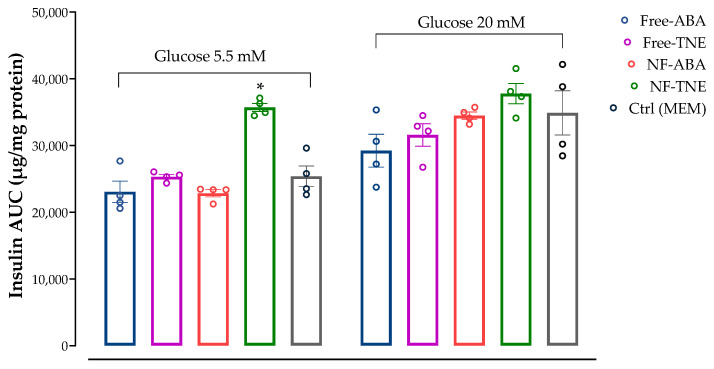
The effect on MIN6 cell insulin secretion after incubation with two different glucose concentrations (5.5 mM and 20 mM). Unformulated abscisic acid (free ABA), unformulated thinned nectarine extract (free TNE), ascorbyl palmitate/DSPE-PEG nanoformulated abscisic acid (NF-ABA), ascorbyl palmitate/DSPE-PEG nanoformulated thinned nectarine extract (NF-TNE), and MEM (Ctrl) were used as treatments. The data represent the mean ± SEM, *n* = 4. Data were analyzed with one-way ANOVA followed by Tukey’s multiple comparison test; * p ≤ 0.05, significantly different from the Ctrl sample at the same glucose concentrations. Abbreviations: AUC, area under the curve; Ctrl, control.

As shown in Figure 7, cells incubated with physiological glucose levels (5.5 mM) showed a significant increase in insulin AUC when incubated with NF-TNE (*p <* 0.05 vs. Ctrl group), resulting in a 40% increase in the AUC value compared with the Ctrl group. In contrast, the other treatment groups showed no significant effects on insulin secretion in this cellular model. A similar trend can be observed in cells incubated with supraphysiological glucose levels (20 mM), as treatment with each of the samples tested showed no significant changes in insulin AUC, although a slight increase was observed in the NF-TNE group (+8.2% vs. Ctrl group). 

Furthermore, if we consider insulin secretion at the various sample collection time points (Figure S4), incubation with both 5.5 mM and 20 mM glucose concentrations resulted in a biphasic insulin secretion profile. The two peaks of secretion are observed at T5 and T60 min. These two peaks may be explained in a physiological context by the rapid release of preformed insulin from cellular granules docked at the periphery of the cells, and the subsequent release of insulin granules that were newly synthesized and more gradually transported from within the cell organelles to exocytosis [27]. In addition, NF-TNE showed a statistical increase in insulin secretion at both T5 and T60 min in cells incubated with 5.5 mM glucose, (*p <* 0.001 vs. Ctrl), or only at T5 when cells were incubated with 20 mM glucose (*p <* 0.01 vs. Ctrl, Figure S4). In contrast, a significant reduction (*p <* 0.001) in insulin release at T60 was observed in the free ABA group compared with the Ctrl group in response to 20 mM glucose exposure. 

### 3.7. Assessment of Insulin Secretion upon Iron Challenge 

**Figure 8:**
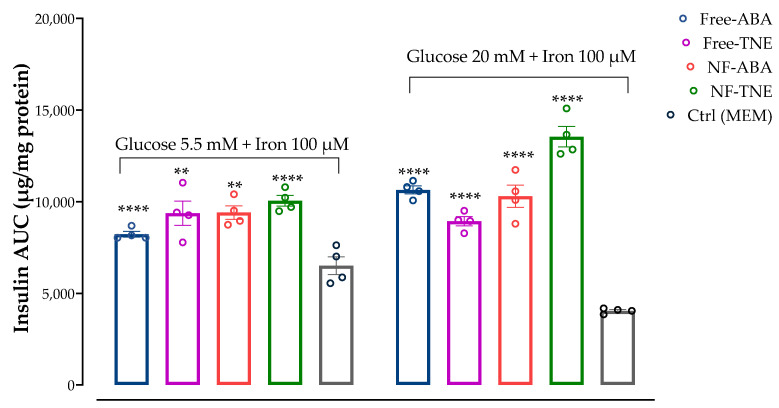
The effect on MIN6 cell insulin secretion after incubation with two different glucose concentrations (5.5 mM and 20 mM) and high iron levels (100 μM). Unformulated abscisic acid (free ABA), unformulated thinned nectarine extract (free TNE), ascorbyl palmitate/DSPE-PEG nanoformulated abscisic acid (NF-ABA), ascorbyl palmitate/DSPE-PEG nanoformulated thinned nectarine extract (NF-TNE), and MEM (Ctrl) were used as treatments. The data represent the mean ± SEM, *n* = 4. Data were analyzed with one-way ANOVA followed by Tukey’s multiple comparison test; ** p ≤ 0.01, **** p ≤ 0.0001 significantly different from the Ctrl sample at the same glucose concentrations. Abbreviations: AUC, area under the curve; Ctrl, control.

Interestingly, as shown in Figure 8, significantly higher insulin AUC values were observed for all test samples compared with the Ctrl group in both experiments that included preincubation with high iron levels (100 μM). Notably, the highest insulin response was obtained in the NF-TNE group, exhibiting 54% higher insulin levels compared with the Ctrl group (*p <* 0.0001). Similar to results from the GSIS experiment, NF samples showed a greater effect on insulin release compared to unformulated samples. Another interesting result is related to cells incubated with both high glucose and iron concentrations. In this regard, significantly higher insulin secretion was observed at the same significance level for all test groups compared with the Ctrl group (*p <* 0.0001 vs. Ctrl group). Moreover, these results are consistent with the observations in the previous section, as the highest insulin concentration was achieved in the NF-TNE group (+235% vs. Ctrl group). 

In terms of insulin response at different analysis time points (Figure S5), a detrimental effect caused by iron incubation can be clearly observed in our cellular model, as only a slight increase in insulin response or a flat-shaped insulin profile was observed in Ctrl cells incubated with the combination of iron and 5.5 mM glucose or 20 mM glucose, respectively. Nevertheless, as shown in the upper graph of the mentioned Figure, insulin secretion at T5 (*p* < 0.001) significantly increased in the free ABA group compared to the Ctrl group, while a clear trend of increase over the incubation period was observed for all the treatment groups, with a significant increase at T60 (*p <* 0.01, *p <* 0.001, *p <* 0.0001 and *p <* 0.0001 for the free ABA, free TNE, NF-ABA, and NF-TNE groups, respectively, compared with Ctrl group). Finally, following incubation with high glucose and iron levels, a significant increase in insulin levels was detected for all the analysis time points in the NF-TNE group, or only at T30 and T60 for the other treatment groups, compared to the Ctrl group (Figure S5, lower graph).

## 4. Discussion

This study aimed to develop, for the first time, nanoformulations containing pure ABA or a TNE at the same ABA concentration. Additionally, the antioxidant and insulinotropic potential of these nanoformulated samples and their unformulated forms were tested and compared in a pancreatic β-cell model. The first set of experiments involved the assessment of cell viability after treatment with testing samples at three different ABA concentrations (0.001, 0.01, and 0.1 µM) at 24, 48, and 72 h. The time points represent short-term to extended chronic exposure, while the concentrations were selected based on prior work [28]. In addition to assessing any potential cytotoxic effects of the samples, another objective of these experiments was to determine the optimum concentration range to be used in further cell exposure experiments involving NFs and free ABA/TNE. An interesting observation was that free TNE increased MIN6 cell viability at all concentrations tested at the initial 24 h time point (Figure 1). One probable explanation may be that TNE contains a complex blend of bioactive compounds in addition to ABA, and it is possible that at the initial time point, these act in concert at the tested concentration range to evoke an acute proliferative effect on cell viability, which is then gradually reduced over time [9]. Furthermore, as reported in the same Figure, a clear trend of increase in MIN6 cell survival was observed over the 72 h incubation period in both the NF-ABA and NF-TNE groups at almost all the concentrations tested, compared to the Ctrl group. Similarly, the increased cell viability observed in the NF samples was significant compared to the free samples at the same ABA concentrations (Figure 2, *p* < 0.05). One possible explanation could be that free compounds in their native, unformulated form evoke rapid effects on intracellular organelles, thereby compromising cell viability to some extent. In the case of formulated delivery systems of standards and extracts, the carrier system may facilitate a more gradual and temporal release of its payload into the intracellular environment upon cellular entry [18]. In this way, the observed effects may be sustained and gradually mitigate any potential adverse effects that occur within the cells following sudden exposure to high levels of bioactive molecules. In addition, the specific composition of the NF may influence cell viability. In this regard, ascorbyl palmitate, a stable form of ascorbic acid containing palmitic acid was used as an excipient for NF preparation. The presence of the fatty acid palmitic acid may also present a nutrient for cells over the 72 h incubation, therefore promoting their eventual proliferation [6]. In summary, the cytotoxicity results demonstrate the suitability of NFs for further cellular experiments and provide information about the concentrations of ABA that could be utilized in the experiments. Doubtless, the obtained results have also revealed intriguing trends that call for future more comprehensive studies to further investigate this phenomenon.

The antioxidant potential of pure ABA and TNE at the same ABA concentration was evaluated and compared in the MIN6 pancreatic line by performing the CAA assay. Since the cellular antioxidant activity of a bioactive compound depends on its cellular uptake, this assay also indicates the relative cellular uptake potential of active molecules from the free extract compared to NFs, as well as the differences between the formulations. Interestingly, neither of the ABA-based groups showed the ability to counteract oxidative stress in our assay, while the results from treatment with TNE demonstrated the ability of this vegetal extract to exert antioxidant properties when cells endure oxidative stress. However, NF-TNE showed significantly higher antioxidant activity compared to free TNE (*p* < 0.001, Figure 3). This result could be explained in part based on the rationale that the unformulated compounds had compromised antioxidant activity before and upon entry into the cellular environment. As explained earlier, many potent natural compounds, most notably curcumin, suffer from issues of stability and degradation that affect their potency [18]. A formulation strategy such as the one used in this study not only preserves the stability and integrity of bioactive compounds but can also improve cellular delivery. In this regard, the DSPE-PEG-AP delivery system used in this study has previously been shown to significantly increase the cellular delivery of iron as the payload in human Caco-2 intestinal cells [21]. Thus, the higher antioxidant activity may be attributed in part to the better preservation of antioxidant activity as well as enhanced cellular delivery, highlighting the beneficial potential of this approach. Furthermore, the components of the NF system retain their antioxidant activity, which may also have influenced cellular antioxidant behavior. As indeed reported in the scientific literature, the ascorbyl moiety of ascorbyl palmitate nanocarrier has been demonstrated to exert even much higher antioxidant activity than ascorbic acid itself [22]. This observation further strengthens the beneficial potential of NF-TNE, as it contains different bioactive compounds that could act synergistically to exert significant cellular antioxidant activity.

In the GSIS and iron-challenge assays, we aimed to evaluate the insulin response on MIN6 cells incubated with two different glucose concentrations, which were chosen to replicate physiological and supraphysiological glucose levels (5.5 and 20 mM respectively), w/wo preincubation with high iron levels (100 μM). The prooxidant role of excess free iron and its detrimental consequences have been demonstrated in various organ systems and disease states. Elevated iron levels have been shown to correlate with the pathogenesis of type-2 diabetes mellitus (T2DM) [29], and previous studies on MIN6 cells have demonstrated 100 μM iron to cause cellular and mitochondrial dysfunction [6]. Pre-treatment with one of the test samples (free ABA, free TNE, NF-ABA, and NF-TNE) or MEM (Ctrl) was then evaluated under the above-described conditions. The initial observation relates to the significant increase in insulin AUC of the Ctrl group incubated with 5.5 mM glucose compared with the same group incubated with higher glucose levels (*p <* 0.05, Figure 6). This is consistent with evidence from the literature on the insulin profile upon MIN6 cells’ exposure to the same glucose levels. In this regard, a progressive response starting at 5 mM glucose has been reported for this cell line, reaching its maximum at 25 mM and remaining at this level up to 50 mM [30]. Moreover, a significant decrease in insulin secretion was observed for both Ctrl groups incubated with 5.5 and 20 mM glucose in combination with 100 µM iron, compared to the same groups without iron preincubation. The selection of iron dose for our experimental design was based on evidence obtained from a previous work performed on the same cellular line [6]. The mentioned study compared the effects of two different iron concentrations, corresponding to 20 µM and 100 µM, on MIN6 β-cells. The study revealed that although both concentrations increased cellular iron accumulation, exposure to 100 µM iron led to a more pronounced increase in lipid peroxidation and a greater reduction in insulin secretion and cellular insulin content. By increasing mitochondrial iron transport, the overaccumulation of this element in β-cells may in fact be responsible for the dysfunction of the mitochondria and the impairment of insulin secretory machinery [31,32]. Moreover, the study reported a significant reduction in the expression of SNAP-25, a key protein in the core SNARE (soluble N-ethylmaleimide-sensitive factor attachment protein receptor) complex responsible for insulin exocytosis, after cell incubation with high iron levels [6]. Accordingly, as shown in Figure 6, if we consider Ctrl cells exposed to both high glucose and iron levels, an even lower level of insulin AUC can be observed compared to cells incubated with 5.5 mM glucose and 100 µM iron, probably due to the detrimental combination of glucose and iron-induced toxicity to which these cells were exposed. Therefore, these results further support the validity of using these conditions as a model to investigate β-cell disruption and insulin secretory dysfunction.

In the present work, we chose to evaluate and compare the effects of different samples on insulin secretion, using the same ABA concentration (0.05 µM) for all test treatments. This concentration was selected based on available evidence about the role of this bioactive molecule in pancreatic β-cell lines. Specifically, Bruzzone et al. reported the ability of ABA to significantly increase insulin secretion from RIN-m and INS-1 cells and murine and human pancreatic islets incubated with both low and high glucose levels (i.e., 5.5 and 16.7 mM glucose). This effect was observed after treatment with ABA at a concentration of 0.01 µM, although no statistical differences were observed with higher concentrations of the pure standard (from 0.01 to 10 µM ABA, *p* = 0.6) [28]. In our cellular model, treatment with ABA-containing samples did not show any significant increase in insulin response after incubation with physiological and supraphysiological glucose concentrations (Figure 7). The only exception was represented by the treatment with free and NF-TNE samples, which resulted in a slight increase in insulin secretion compared with the Ctrl group. A completely different observation arises from results obtained in MIN6 cells incubated with both glucose and iron (Figure 8). In this regard, significantly higher insulin AUC levels were observed for all test samples compared with the Ctrl group, with the greatest effect obtained after treatment with NF-TNE (+54% vs. Ctrl group incubated with 5.5 mM glucose + 100 μM iron; +235% vs. Ctrl group incubated with 20 mM glucose + 100 μM iron). As previously reported, the role of excessive iron accumulation in damaging essential biological components through the generation of ROS is well established [3,5,33]. Therefore, ROS clearly have the ability to behave like a destructive agent that eventually leads to insulin resistance and β-cell failure [34]. Notably, the impairment of insulin secretion due to iron-induced oxidative stress can lead to hyperglycemia and insulin resistance conditions, which are well-known features of T2DM. In this context, recent studies showed that iron excess negatively affected insulin activity [5,29,35]. In these considerations, the presence of bioactive compounds with antioxidant potential, especially polyphenols, in the TNE-based sample has been shown to contribute to the protection of these cells by counteracting the cellular dysfunction induced by ROS in our model. Moreover, in both experiments involving exposure to high iron levels, NF samples were found to be more efficient in terms of insulin release compared to unformulated samples. These data are also supported by the results of our cell viability and antioxidant activity assays, where the NF samples generally demonstrated prominent positive effects. Therefore, this evidence further supports the advantages of using a formulation-based approach for the delivery of bioactive molecules [18,19,21]. 

To better understand the different results obtained with the incubation with NF-ABA, NF-TNE, ABA, and TNE, it is important to consider both the effects of nanoformulations and the distinct nature of thinned nectarine extract compared to pure ABA. The composition of thinned nectarine extract (TNE) differs fundamentally from that of pure ABA [9]. In this regard, TNE contains a complex mix of phytochemicals, including polyphenols, which may synergistically contribute to its biological activities [9,36]. This complexity can result in a variety of interactions with cellular components and signaling pathways, in contrast to the singular interaction of pure ABA. Moreover, nanoformulations significantly modify the properties of bioactive compounds, potentially enhancing their stability, solubility, and bioavailability [37]. More specifically, nanoformulated compounds might demonstrate improved cellular delivery and efficacy of various bioactive substances, leading to more pronounced biological effects [20,38]. Nanoformulations might also change how active ingredients interact with cellular receptors or signal transduction pathways [39]. For example, alterations in the molecular size or surface properties of nanoformulations could affect recognition by cellular receptors, thereby influencing signal transduction [40]. Therefore, the differences between nanoformulated and non-nanoformulated ABA and TNE can be attributed to both the changes in the physicochemical properties of the nanoformulated samples and the complex nature of TNE compared to pure ABA. However, more specific mechanistic insights into signal transduction and receptor interactions would require further experimental studies.

The originality of the present project lies in the first-time evaluation and comparison of the TNE efficacy on pancreatic β-function at the same ABA concentrations of a pure standard and the first-time incorporation of these molecules into formulations using novel delivery systems. Specifically, the evaluation at the same concentration of the active ABA allowed us to distinguish the additional activity of TNE in relation to its content of bioactive compounds, especially polyphenols, which can contribute positively and synergistically to cellular antioxidant protection.

## 5. Conclusions

In conclusion, two NFs containing pure ABA or TNE at the same ABA concentration were developed for the first time and optimized using ascorbyl palmitate/DSPE-PEG as the nanocarrier delivery system. Overall, we herein showed the higher efficacy of the tested nanoformulated samples in terms of cell viability, antioxidant activity, and insulin secretion in our pancreatic β-cell model. Furthermore, the improved results obtained in cells treated with the NF-TNE sample support its additional potential to enhance cellular antioxidant defense due to the synergistic combination of bioactive compounds contained in the TNE phytocomplex. Taken together, the obtained results demonstrate not only the unique efficacy of TNE as a bioactive agent with therapeutic potential but also a significant advantage of using a nanoformulation approach to further increase the benefits of this and similar phytobioactive molecules. This novel work lays the foundation for future studies in cellular models to further clarify the mechanisms underlying these observed effects and progress to subsequent human clinical trials.

## Figures and Tables

**Table 1:** Particle size and zeta potential of ABA and TNE-based nanoformulations.

Sample	Particle Size (nm)	Zeta Potential (mV)
DSPE-PEG-AP-ABA	52 ± 3.2	−0.01 ± 0.04
DSPE-PEG-AP-TNE	24 ± 2.1	0.03 ± 0.04
DSPE-PEG-AP-BLK	173 ± 59	−5.3 ± 4.4
DSPE-PEG-ABA	5.2 ± 1.3	−0.001 ± 0.03
DSPE-PEG-TNE	6.2 ± 0.7	0.0051 ± 0.002
DSPE-PEG-BLK	3.6 ± 1.5	0.0861 ± 1.5

Data represent the mean ± standard deviation (SD) of three replicates. Abbreviations: ABA, abscisic acid; AP, ascorbyl palmitate; BLK, blank; DSPE-PEG: polyethylene glycol-grafted 1,2-distearoyl-sn-glycerol-3-phosphatidylethanolamine; TNE, thinned nectarine extract.

## Data Availability

Data are contained within the article and Supplementary Materials.

## Supplementary Materials

The supporting information can be downloaded at: https://www.mdpi.com/article/10.3390/antiox13010063/s1, Table S1. List of samples at different glucose and iron concentrations; Figure S1. Representative image of DSPE-AP nanocarriers imaged at 13,500 magnification; Figure S2. Growth pattern of MIN6 cells into mature pseudoislets; Figure S3. Cellular antioxidant activity of the positive control Quercetin at different concentrations (0–2000 µM). Values are presented as means ± S.D. of 6 replicates; Figure S4. The effect on MIN6 cells insulin secretion after incubation with glucose 5.5 mM (upper graph) and glucose 20 mM (lower graph); Figure S5. The effect on MIN6 cells insulin secretion after incubation with a combination of glucose 5.5 mM (upper graph) and glucose 20 mM (lower graph) with high iron levels (100 μM).

## References

[1] Eguchi N., Vaziri N.D., Dafoe D.C., Ichii H. (2021). The role of oxidative stress in pancreatic β cell dysfunction in diabetes. Int. J. Mol. Sci..

[2] Benáková Š., Holendová B., Plecitá-Hlavatá L. (2021). Redox homeostasis in pancreatic β-cells: From development to failure. Antioxidants.

[3] Gerber P.A., Rutter G.A. (2017). The Role of Oxidative Stress and Hypoxia in Pancreatic Beta-Cell Dysfunction in Diabetes Mellitus. Antioxid. Redox Signal..

[4] Schieber M., Chandel N.S. (2014). ROS function in redox signaling. Curr. Biol..

[5] Hansen J.B., Moen I.W., Mandrup-Poulsen T. (2014). Iron: The hard player in diabetes pathophysiology. Acta Physiol..

[6] Blesia V., Patel V.B., Al-Obaidi H., Renshaw D., Zariwala M.G. (2021). Excessive Iron Induces Oxidative Stress Promoting Cellular Perturbations and Insulin Secretory Dysfunction in MIN6 Beta Cells. Cells.

[7] Tran N., Pham B., Le L. (2020). Bioactive compounds in anti-diabetic plants: From herbal medicine to modern drug discovery. Biology.

[8] Rao P., Rathod V. (2019). Valorization of Food and Agricultural Waste: A Step towards Greener Future. Chem. Rec..

[9] Schiano E., Piccolo V., Novellino E., Maisto M., Iannuzzo F., Summa V., Tenore G.C. (2022). Thinned Nectarines, an Agro-Food Waste with Antidiabetic Potential: HPLC-HESI-MS/MS Phenolic Characterization and In Vitro Evaluation of Their Beneficial Activities. Foods.

[10] Redondo D., Arias E., Oria R., Venturini M.E. (2017). Thinned stone fruits are a source of polyphenols and antioxidant compounds. J. Sci. Food Agric..

[11] Leng P., Yuan B., Guo Y., Chen P. (2014). The role of abscisic acid in fruit ripening and responses to abiotic stress. J. Exp. Bot..

[12] Sordillo L.M., Russo M.A., Roma D., Shaikh I.S.R., Zocchi E., Hontecillas R., Leber A., Einerhand A., Carbo A., Bruzzone S. (2017). Abscisic Acid: A Novel Nutraceutical for Glycemic Control. Front. Nutr..

[13] Magnone M., Sturla L., Guida L., Spinelli S., Begani G., Bruzzone S., Fresia C., Zocchi E. (2020). Abscisic acid: A conserved hormone in plants and humans and a promising aid to combat prediabetes and the metabolic syndrome. Nutrients.

[14] Egbuna C., Awuchi C.G., Kushwaha G., Rudrapal M., Patrick-Iwuanyanwu K.C., Singh O., Odoh U.E., Khan J., Jeevanandam J., Kumarasamy S. (2021). Bioactive Compounds Effective Against Type 2 Diabetes Mellitus: A Systematic Review. Curr. Top. Med. Chem..

[15] Maritim A.C., Sanders R.A., Watkins J.B. (2003). Diabetes, oxidative stress, and antioxidants: A review. J. Biochem. Mol. Toxicol..

[16] Guo C., Bi J., Li X., Lyu J., Zhou M., Wu X. (2020). Antioxidant profile of thinned young and ripe fruits of Chinese peach and nectarine varieties. Int. J. Food Prop..

[17] Cantín C.M., Gogorcena Y., Moreno M.Á. (2010). Phenotypic diversity and relationships of fruit quality traits in peach and nectarine [*Prunus persica* (L.) Batsch] breeding progenies. Euphytica.

[18] Muthukrishnan L. (2022). Nanonutraceuticals—Challenges and Novel Nano-based Carriers for Effective Delivery and Enhanced Bioavailability. Food Bioprocess Technol..

[19] Mursaleen L., Chan S.H.Y., Noble B., Somavarapu S., Zariwala M.G. (2023). Curcumin and N-Acetylcysteine Nanocarriers Alone or Combined with Deferoxamine Target the Mitochondria and Protect against Neurotoxicity and Oxidative Stress in a Co-Culture Model of Parkinson’s Disease. Antioxidants.

[20] Mursaleen L., Noble B., Somavarapu S., Zariwala M.G. (2021). Micellar nanocarriers of hydroxytyrosol are protective against parkinson’s related oxidative stress in an in vitro hcmec/d3-sh-sy5y co-culture system. Antioxidants.

[21] Zariwala M.G., Farnaud S., Merchant Z., Somavarapu S., Renshaw D. (2014). Ascorbyl palmitate/DSPE-PEG nanocarriers for oral iron delivery: Preparation, characterisation and in vitro evaluation. Colloids Surf. B Biointerfaces.

[22] Moribe K., Limwikrant W., Higashi K., Yamamoto K. (2011). Drug Nanoparticle Formulation Using Ascorbic Acid Derivatives. J. Drug Deliv..

[23] Schiano E., Neri I., Maisto M., Novellino E., Iannuzzo F., Piccolo V., Summa V., Grumetto L., Tenore G.C. (2023). Validation of an LC-MS/MS Method for the Determination of Abscisic Acid Concentration in a Real-World Setting. Foods.

[24] Bosco R., Caser M., Vanara F., Scariot V. (2013). Development of a rapid LC-DAD/FLD method for the simultaneous determination of auxins and abscisic acid in plant extracts. J. Agric. Food Chem..

[25] Wolfe K.L., Rui H.L. (2007). Cellular antioxidant activity (CAA) assay for assessing antioxidants, foods, and dietary supplements. J. Agric. Food Chem..

[26] Pap R., Pandur E., Jánosa G., Sipos K., Fritz F.R., Nagy T., Agócs A., Deli J. (2023). Protective Effects of 3′-Epilutein and 3′-Oxolutein against Glutamate-Induced Neuronal Damage. Int. J. Mol. Sci..

[27] Rorsman P., Eliasson L., Renström E., Gromada J., Barg S., Göpel S. (2000). The Cell Physiology of Biphasic Insulin Secretion. Physiology.

[28] Bruzzone S., Bodrato N., Usai C., Guida L., Moreschi I., Nano R., Antonioli B., Fruscione F., Magnone M., Scarfì S. (2008). Abscisic acid is an endogenous stimulator of insulin release from human pancreatic islets with cyclic ADP ribose as second messenger. J. Biol. Chem..

[29] Shaaban M.A., Dawod A.E.A., Nasr M.A. (2016). Role of iron in diabetes mellitus and its complications. Menoufia Med. J..

[30] Tanabe K., Liu Y., Hasan S.D., Martinez S.C., Cras-Méneur C., Welling C.M., Bernal-Mizrachi E., Tanizawa Y., Rhodes C.J., Zmuda E. (2011). Glucose and fatty acids synergize to promote B-cell apoptosis through activation of glycogen synthase kinase 3β independent of JNK activation. PLoS ONE.

[31] Ma Z.A., Zhao Z., Turk J. (2012). Mitochondrial Dysfunction and β-Cell Failure in Type 2 Diabetes Mellitus. Exp. Diabetes Res..

[32] Marku A., Galli A., Marciani P., Dule N., Perego C., Castagna M. (2021). Iron Metabolism in Pancreatic Beta-Cell Function and Dysfunction. Cells.

[33] Nakamura T., Naguro I., Ichijo H. (2019). Iron homeostasis and iron-regulated ROS in cell death, senescence and human diseases. Biochim. Biophys. Acta-Gen. Subj..

[34] Tiganis T. (2011). Reactive oxygen species and insulin resistance: The good, the bad and the ugly. Trends Pharmacol. Sci..

[35] Aregbesola A., Voutilainen S., Virtanen J.K., Mursu J., Tuomainen T. (2013). Body iron stores and the risk of type 2 diabetes in middle-aged men. Eur. J. Endocrinol..

[36] Mengyuan W., Haoli W., Tingting M., Qian G., Yulin F., Xiangyu S. (2021). Comprehensive Utilization of Thinned Unripe Fruits from Horticultural Crops. Foods.

[37] Han H.S., Koo S.Y., Choi K.Y. (2021). Emerging nanoformulation strategies for phytocompounds and applications from drug delivery to phototherapy to imaging. Bioact. Mater..

[38] Bala S., Garg D., Sridhar K., Inbaraj B.S., Singh R., Kamma S., Tripathi M., Sharma M. (2023). Transformation of Agro-Waste into Value-Added Bioproducts and Bioactive Compounds: Micro/Nano Formulations and Application in the Agri-Food-Pharma Sector. Bioengineering.

[39] Donahue N.D., Acar H., Wilhelm S. (2019). Concepts of nanoparticle cellular uptake, intracellular trafficking, and kinetics in nanomedicine. Adv. Drug Deliv. Rev..

[40] Scheinberg D.A., Grimm J., Heller D.A., Stater E.P., Bradbury M., McDevitt M.R. (2017). Advances in the clinical translation of nanotechnology. Curr. Opin. Biotechnol..

