# Supplementary material for: Antioxidant and Antidiabetic Properties of a Thinned-Nectarine-Based Nanoformulation in a Pancreatic β-Cell Line
Source: Antioxidants (Basel). 2023 Dec 31;13(1):63. doi: 10.3390/antiox13010063 (PMC10812739; doi:10.3390/antiox13010063)
Supplement: Supplementary file 1 [file antioxidants-13-00063-s001.zip › antioxidants-2746410-supplementary.pdf]

SUPPLEMENTARY MATERIALS

Table S1. List of samples at different glucose and iron concentrations.

Treatment	Abbreviation	Glucose Concentration (mM)	Iron Concentration (μM)
DSPE-PEG/AP nanoformulation of TNE	NF-TNE 1	5.5	0
	NF-TNE 2	20	0
	NF-TNE 3	5.5	100
	NF-TNE 4	20	100
DSPE-PEG/AP nanoformulation of ABA	NF-ABA 1	5.5	0
	NF-ABA 2	20	0
	NF-ABA 3	5.5	100
	NF-ABA 4	20	100
Free nanocarriers	BLK-NF 1	5.5	100
	BLK-NF 2	20	100
Free TNE	Free-TNE 1	5.5	0
	Free-TNE 2	20	0
	Free-TNE 3	5.5	100
	Free-TNE 4	20	100
Free ABA	Free-ABA 1	5.5	0
	Free-ABA 2	20	0
	Free-ABA 3	5.5	100
	Free-ABA 4	20	100
Media (MEM)	Ctrl-Glu 1	5.5	0
	Ctrl-Glu 2	20	0
	Ctrl-Iron 1	5.5	100
	Ctrl-Iron 2	20	100

Abbreviations: ABA, abscisic acid; AP, ascorbyl palmitate; BLK, blank; DSPE-PEG: Polyethylene glycol grafted 1,2-distearoyl-sn-glycerol-3-phosphatidylethanolamine; NF, nanoformulated; TNE, thinned nectarine extract.

Figure S1. Representative image of DSPE-AP nanocarriers imaged at 13,500 magnification.

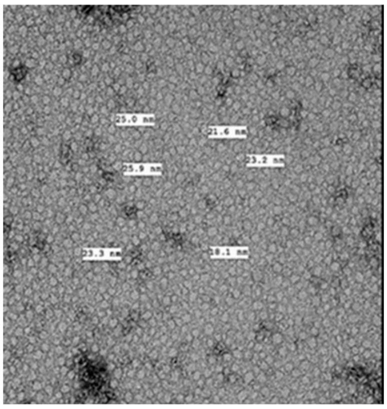

Figure S2. Growth pattern of MIN6 cells into mature pseudoislets.

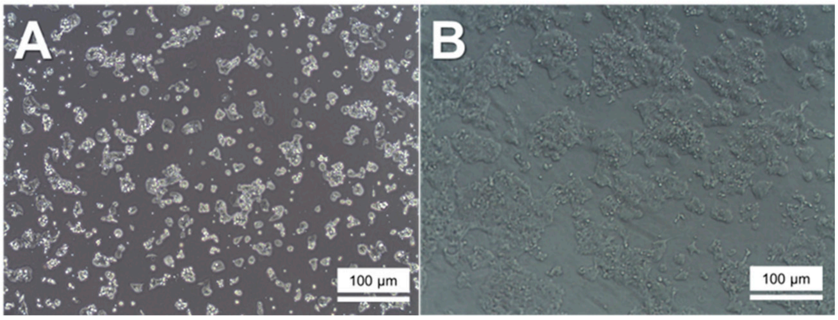

Stock MIN6 cell cultures were grown at 37°C in 75 cm2 T-flasks, replacing the medium every two days. Cells were seeded in 12-well plates with a density of 75 x 104 cells/cm2 for all experimental cultures (panel A). MIN6 cells reached confluence on day 5 post-seeding by which point the phenotype of small clusters of cells (pseudoislets) was formed, indicating the cells were functional and capable of secreting insulin (panel B). Cells were imaged through a microscope at 10x magnification.

Figure S3. Cellular antioxidant activity of the positive control Quercetin at different concentrations (0-2000 µM). Values are presented as means ± S.D. of 6 replicates.

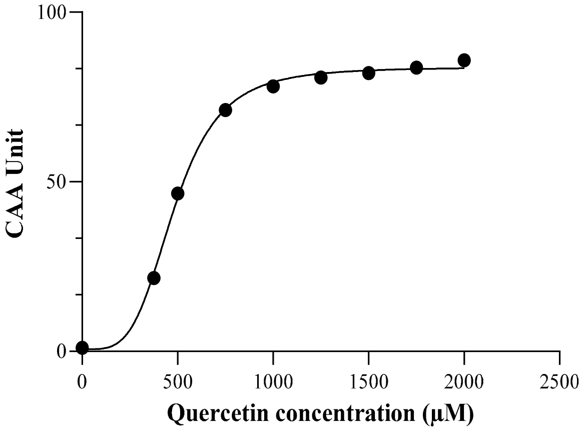

Figure S4. The effect on MIN6 cells insulin secretion after incubation with glucose 5.5 mM (upper graph) and glucose 20 mM (lower graph).

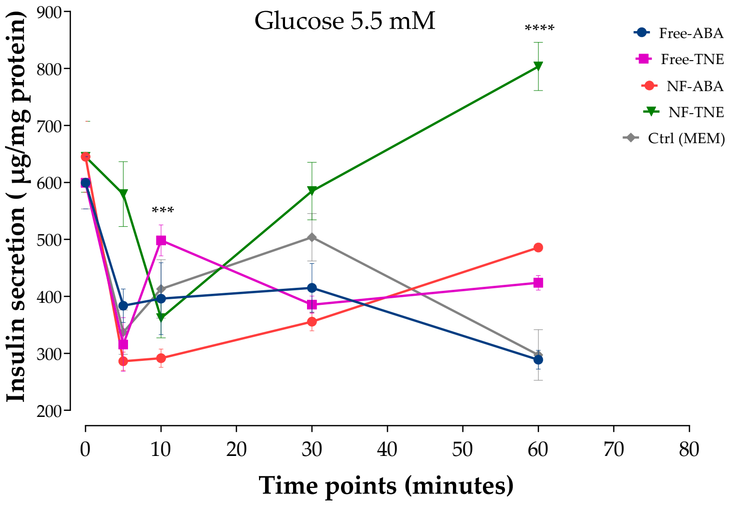

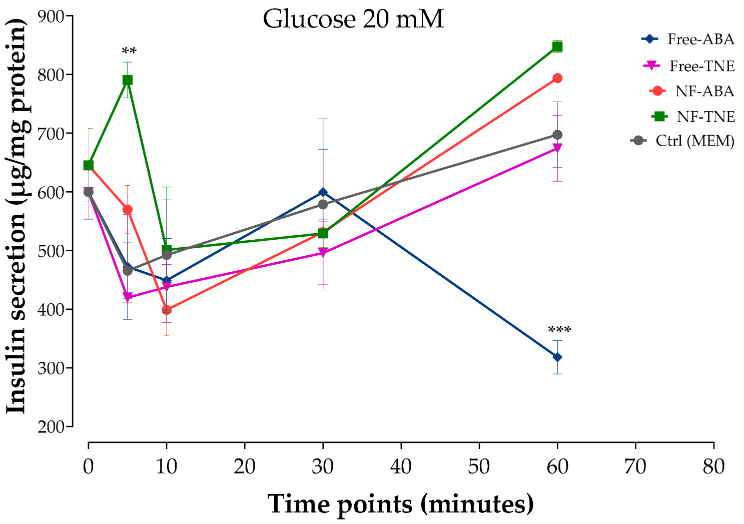

Insulin secretion was evaluated at different time points of analysis (T0, T5, T10, T30, and T60 minutes). Unencapsulated abscisic acid (Free-ABA), unencapsulated thinned nectarine extract (Free-TNE), ascorbyl palmitate/DSPE-PEG nanoformulated abscisic acid (NF-ABA), ascorbyl palmitate/DSPE-PEG nanoformulated thinned nectarine extract (NF-TNE) and MEM (Ctrl) were used as treatments. The data represent mean ± SEM, n = 4. Data were analyzed with two-way ANOVA followed by Tukey's multiple comparison test; $*p \leq 0.05$, $**p \leq 0.01$, $***p \leq 0.001$, $****p \leq 0.0001$ significantly different from Ctrl sample (MEM) at the same time point of analysis.

Figure S5. The effect on MIN6 cells insulin secretion after incubation with a combination of glucose 5.5 mM (upper graph) and glucose 20 mM (lower graph) with high iron levels (100 µM).

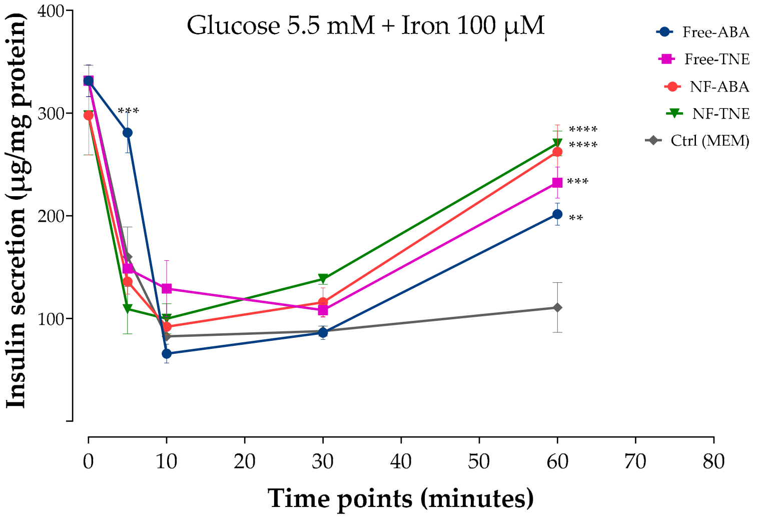

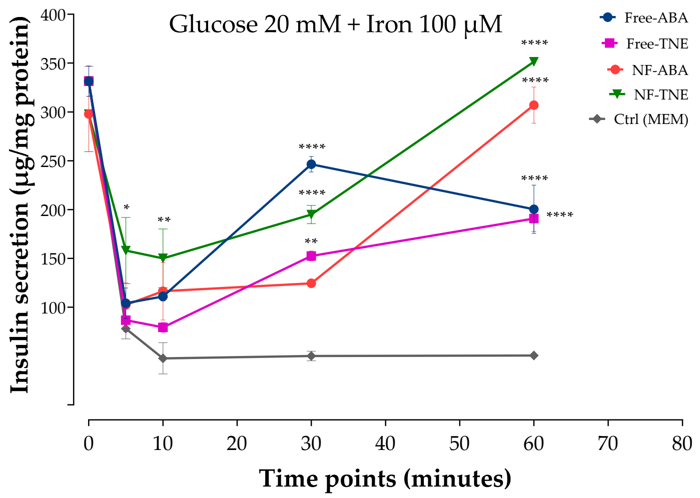

Insulin secretion was evaluated at different time points of analysis (T0, T5, T10, T30, and T60 minutes). Unencapsulated abscisic acid (Free-ABA), unencapsulated thinned nectarine extract (Free-TNE), ascorbyl palmitate/DSPE-PEG nanoformulated abscisic acid (NF-ABA), ascorbyl palmitate/DSPE-PEG nanoformulated thinned nectarine extract (NF-TNE) and MEM (Ctrl) were used as treatments. The data represent mean ± SEM, n = 4. Data were analyzed with two-way ANOVA followed by Tukey's multiple comparison test; *$p \le 0.05$, **$p \le 0.01$, ***$p \le 0.001$, ****$p \le 0.0001$ significantly different from Ctrl sample (MEM) at the same time point of analysis.
